# Editorial: Rising stars in hematopoiesis and stem cells 2023

**DOI:** 10.3389/frhem.2024.1477997

**Published:** 2024-08-29

**Authors:** Marcus O. Muench, Kristbjorn Orri Gudmundsson

**Affiliations:** 1 Vitalant Research Institute, San Francisco, CA, United States,; 2 Departments of Pathology and Laboratory Medicine, University of California, San Francisco, San Francisco, CA, United States,; 3 Center for Advanced Preclinical Research, Frederick National Laboratory for Cancer Research, Center for Cancer Research, National Cancer Institute, National Institutes of Health (NIH), Frederick, MD, United States

**Keywords:** hematopoietic stem & progenitor cells (HSPCs), metabolism, extramedullary hematopoiesis, blood pharming, transfusion, asymmetric cell division, fate decisions

We are pleased to present four investigators as our Rising Stars in Hematopoiesis and Stem Cells for 2023. These investigators have recently reviewed, in their respective areas of expertise, recent findings and current research directions in Frontiers in Hematology.

Rattigan is a research associate at the University of Glasgow, Scotland, UK where he has made important discoveries on the metabolic vulnerabilities of chronic myeloid leukemia stem cells. In recent years, themetabolic requirements of cancer cells have been described and specific metabolic dependencies identified could offer novel therapeutic opportunities. In his review, Rattigan discusses the importance of going beyond the reductionist approach, where metabolism is predominantly analyzed at the organelle level, and instead, also focus on the whole organism since targeting metabolic pathways in leukemia will have systemic effects. Importantly, he reviews the challenges facing biomedical researchers in identifying and targeting leukemia-specific metabolic pathways without affecting the function of normal cells since there is significant overlap in the utilization of these pathways. Finally, Rattigan discusses the gains that have been made in the clinic in metabolic pathway targeting in leukemia and the new metabolic vulnerabilities that may be targeted in the future.

Rivera-Torruco is a post-doctoral scholar at Vitalant Research Institute studying developmental hematopoiesis. She reviews the topic of extramedullary hematopoiesis during prenatal development as well as in adults. A focus of this review is her work on isthmin-1, a marker of hematopoietic progenitors in the mouse lung (1). As a graduate student, she performed this work at Hospital Infantil de México Federico Gómez, Universidad Nacional Autónoma de México in Mexico. Some of the unique properties of extramedullary hematopoiesis during early ontogeny and its role in adult hematopoiesis will surely be studied for years to come.

Hassan reviews the current state of blood pharming–the effort to produce blood cells in culture for transfusion. Hassan, an assistant professor at the Centre for Regenerative Medicine and Stem Cell Research at the Aga Khan University in Pakistan, studies erythropoiesis and the technologies needed to generate red cells *in vitro* in quantities sufficient to be used for blood transfusion. He reviews the different sources of stem cells being tested as feedstock for red cell cultures as well as the many challenges, both technical and financial, that need to be overcome to make blood pharming a reality. This challenge, which tests our basic knowledge of erythropoiesis and hematopoiesis in general, has the potential to address blood shortages and safety concerns. It also offers opportunities to produce bespoke red cells or red cells with unique therapeutic properties.

Loeffler is a principal investigator at the Department of Hematology, St. Jude Children’s Research Hospital where he focuses on deciphering the molecular mechanism of hematopoietic stem cell (HSC) self-renewal and fate decisions. Loeffler has made major discoveries in stem cell biology by demonstrating, through lysosomal tracking, that HSCs divide asymmetrically. Importantly, he has shown that the asymmetric inheritance of cellular organelles leads to different cellular fates for stem cell progeny. In this review, Nunes and Loeffler give a historical overview of asymmetric cell division research and discuss previous findings and debates on the mechanism of HSC asymmetric cell division. He presents direct evidence supporting asymmetric cell division in HSC and describes the role of lysosomal and mitochondrial asymmetric inheritance in HSC fate decisions as well as the potential role of other cellular organelles in this process. Understanding these mechanisms is important in the quest for successful HSC expansion protocols for therapeutic purposes.

We encourage readers of Frontiers in Hematology to pay attention to the works of these four rising stars in the field of hematopoiesis and stem cells. We also look forward to their many contributions in the future.
